# Report on the Epidemic Cholera at Buffalo, in 1849

**Published:** 1849-11

**Authors:** 

**Affiliations:** Buffalo


### BUFFALO MEDICAL JOURNAL

AND

### MONTHLY REVIEW.

VOL. 5. NOVEMBER, 1849. NO. 6.

### ORIGINAL COMMUNICATIONS.

ART. I.—*Report on the Epidemic Cholera at Buffalo, in* 1849. By the EDITOR.

The first case of Epidemic Cholera reported, as such, by the Board of Health of this city, occurred on the 30th of May, 1849. Previously to this date we had no knowledge of any cases suspected to be cases of cholera, with the exception of a single case, a few days before, which was probably a case of cholera, but the evidences were not considered by the medical attendants sufficiently positive to authorise its being reported as such. The patient arrived at this port from the west, in a steamer, late in the disease, and died at the Hospital of the Sisters of Charity.

The Epidemic had, for some weeks, existed at various places on the Mississippi and Ohio rivers,—Cincinnati, St. Louis, etc.,—and a short time previously had made its appearance at Chicago.

The first reported case occurred on board a steamer from Chicago, from which the patient was transferred to a hotel, before the character of the disease was ascertained.

The second reported case also occurred on board a steamer, from Sandusky, the patient being *en route* from Cincinnati. This case was reported on June 1st.

June 4th, the first indigenous case was reported by the Board. This case occurred in a part of the city distant about a mile and a half from the central street (Main St.) in a north-westerly direction, (in the neighborhood of the work house). The patient was a female, and the case could have had no possible connection (moral or physical) with the cases that had previously occurred.

The fourth case, on the 4th of June, was on board a steamer from Chicago. The patient had been attacked with the disease before the vessel reached this port, and was quite, or nearly convalescent on her arrival, so that she left town in two or three days on her journey eastward.

The fifth case reported by the Board, was on the 8th of June. This occurred in Norton street, a street situated near the ship canal, connecting the creek, or harbor, with the Erie canal. This was the *second* indigenous case.

On the 9th of June, two cases occurred. The subjects were the wife and child of a respectable Irish mechanic, on his way from Brooklyn, L. I., westward.

The eighth reported case was on the 11th of June, and occurred at Younglove's tavern, on Seneca street. This was the *third* indigenous case, and the place of its occurrence was in a part of the town opposite to that of either of the preceding cases originating in the city; nor had the patient been brought into contact with any of the patients previously affected with the disease.

Seven other cases were reported on the same date, (11th). Of these, two occurred on board a canal boat; two in Peacock street, situated near the canal; one in Bennet street, situated in a north-easterly part of the town, remotely distant from all the places in which the previous cases had occurred; one in Norton street, where one case had already occurred; and one in Court street, considerably removed from the sites of the preceding cases.

On the 14th of June, seven cases were reported. Three of these occurred in Rock St., a street situated near the canal, and of a character analogous to that of the five points in the city of New York. One case occurred in Mohawk St., a street quite distant from any of the others in which the disease had existed. One case at the American Hotel, in the centre of the city; and in two cases, the residences of the patients were not stated. These seven cases occurred between the 11th and 14th of June, the Board of Health holding sessions only on those days.

Between the 14th and 20th of June, (the Board meeting again on the latter date), six cases had occurred. Of these, five were in Rock street, and one in Fifth street, a street also distant from those in which the previous cases had been developed.

On the 23d of June, at the next meeting of the Board, six cases were reported. Of these, *one* case occurred in Erie street, *one* in Green street, *one* in Rock street, *one* in Clinton street, and in *one* the street was not stated. These localities are separated from each other by distances of, from a quarter, to half a mile, with the exception that Rock street intersects Erie street. The residences of the patients, in the reports, are not given with more exactness than the names of the streets.

On the 25th of June, five cases were reported; *one* in Rock street, *one* in Erie street, *one* on corner of Eagle and Main streets, (a new site), *one* on the steamer Globe, and *one* on the steamer Niagara.

We have not deemed it important to examine the proceedings of the Board of Health farther, in order to note the different streets in which cases subsequently occurred. The reader will, at once, perceive the reason of our having done so thus far. This particularity in the history of the rise and progress of the epidemic, has reference to the question of contagion—a question not yet settled to the satisfaction of all minds. We waive for the present any remarks on this subject.

From the date last mentioned, June 25, the Epidemic continued to increase. The Board of Health held daily sessions, and issued daily bulletins, the number of cases steadily increasing from week to week, (with daily fluctuations), up to July 24th, when a larger number of cases were reported than on any other day during the existence of the Epidemic. On that day the number of cases reported to the Board was 103, of deaths 32. From this date, the prevalence of the disease decreased, the number of cases steadily and gradually diminishing on each week, up to Sept. 7th, when the Board ceased to issue their daily bulletins. Some cases have since occurred, and at the present time, (Sept. 29th), the disease is not entirely extinct.* As an Epidemic, however, it cannot now be said to prevail, all apprehension of the disease in the public mind has disappeared, and business has again resumed its usual activity.

The whole number of cases reported to the Board of Health from May 30th, to Sept. 7th, was 2,505; number of deaths during the same period, 858, making a ratio of mortality as 1 to a fraction less than 3. The question may arise, in how far the reports to the Board of Health are to be relied upon, as embracing, in the first place, veritable cases of the Epidemic, and, in the second place, comprehending all, or the greater portion of cases which occurred. As respects the first point, the Profession of the city were united in their views of the criteria necessary to the diagnosis of the disease, and, we believe, as a general remark, were careful to recog-

* Oct. 10.—No cases have come to our knowledge since the 29th ult.

nise as cases of the Epidemic, only those in which the evidence was unequivocal. Moreover, the Board of Health, consisting of three members, at first embraced two medical gentlemen, Drs. Haddock and Barnes, who, in conjunction with the Health Physician, Dr. J. S. Trowbridge, decided on the merits of cases of a doubtful character, frequently rejecting cases which were obviously deficient in diagnostic traits.* Still, it is altogether probable that some cases may have been accepted by the Board, which were properly only cases of cholerine, or the diarrhœa whlch generally precedes cholera, and, during the prevalence of the Epidemic, affects large numbers without eventuating in the disease. The fact that but few irregular practitioners were in the habit of reporting cases, renders the records of the Board much more reliable, as respects the point under remark, than they would otherwise have been. In some places, as is well known, cholera reports have included a host of spuri.us cases, some reported through ignorance, by incompetent practitioners, and more (for selfish purposes) through design, by empirics. Such was the case, if at all, in but a limited degree, in Buffalo. The rigid scrutiny pursued by the medical gentlemen in the Board, and the Health Physician, in the investigation of cases of a doubtful character, or emanating from suspicious quarters, at the commencement of the Epidemic, prevented fraudulent efforts, such as have been practiced in other places, to secure a meretricious reputation for extraordinary success in the treatment of the disease. The irregular Faculty of this place, moreover, is so heterogeneous in its composition, that an union for the purpose of effecting an organization of the Board of Health to promote their objects, as was accomplished in Cincinnati, would hardly be practicable. Even the Homœopathic branch of empiricism is divided into factions, sustaining toward each other somewhat of the relation of Kilkenny cats. The great mass of the community, too, whatever the intentions of many might have been at other times, as respects the different species of illegitimate medicine, were reluctant to place their dependence upon any of them at this juncture. These circumstances are cited in explanation ot the fact that few cases were reported by other than regular Physicians, and that the reader may form some opinion of the reliance to be placed on the reports issued by the Board of Health, as containing only veritable cases of cholera.

The second inquiry, viz , if these reports included all, or nearly all the

* Dr. Haddock, a regularly educated physician, but not a practitioner, and a highly respectable citizen, fell a victim to the disease, shortly after the commencement of the Epidemic. The vacancy was not filled by a physician, but Dr. Barnes continued a member of the Board from the commencement of the Epidemic until its close.

cases that occurred, cannot be answered affirmatively. We believe that Physicians were generally faithful in reporting all their cases, but omissions would, doubtless, sometimes occur from inadvertency, or negligence, at a time of such constant and pressing professional duties. Some persons, it is known, died without receiving any medical attendance. Some were attended by irregular practitioners, who reported few, if any cases. The absence of any registration of deaths in this city, rendered it difficult to supply, with any exactness, the defects of the reports of fatal cases by the Board of Health. It is only known that, in many instances, persons died with the disease who were not reported. With respect to cases not ending fatally, we have no means whatever of forming any idea as to the degree in which the reports of the Board of Health represented inadequately the prevalence of the disease. We can only say, that it is by no means probable they included *all* the cases that occurred, but it is probable that they are to be considered as a fair approximation to completeness in this particular.

The disease was not restricted in its prevalence to any particular sections, but was diffused in greater or less degree, over the whole city. A predilection for some localities, however, was manifested by the occurrence of a greater proportion of cases than in other portions. The parts of the city where cases were most numerous, are confessedly less salubrious than those in which the disease prevailed in a less degree, and, at the same time, contained a larger proportion of the classes of population furnishing (as statistics show) the larger number of victims. The sections in which the Epidemic prevailed most, and with the greatest virulence, were, 1st, streets in close proximity to the canal, densely inhabited, and abounding most in poverty and vice; 2d, a part of the city, known as the Hydraulics, in which miasmatic affections are somewhat rife, and the population, generally speaking, not of a character to be peculiarly exempt from an epidemic disease; and, 3d, in a part lying in a north-eastern direction from the centre of the city, in which the population consists, almost wholly, of German laborers. That the disease predominated in these three sections is well known; but we have no data for determining the numerical preponderance with any exactness. In the reports of Physicians to the Board of Health, generally the street alone was designated, in which cases occurred. This is quite indefinite as to the particular situation of the residences of patients, as most of the streets are long, many of them several miles in length.

In order to give some idea of the diffusion of the disease, and as a matter of interest to those of our readers who are familiar with the topography of the city, we have procured a list of the streets in which deaths occurred, and the number of deaths which occurred in them respectively, as follows:

| | | | | | |
|---|---|---|---|---|---|
| Mississippi street, | 12 | Michigan street, | 23 | Clinton street, | 8 |
| Peacock " | 9 | Elk " | 14 | State " | 7 |
| South Division " | 2 | Oak " | 19 | Tupper " | 6 |
| Canal " | 3 | Scott " | 5 | Commercial " | 3 |
| Vine " | 4 | Seneca " | 16 | Eagle " | 9 |
| Swan " | 3 | Batavia " | 8 | Delaware " | 1 |
| North Division " | 6 | Cypress " | 2 | Virginia " | 5 |
| Lloyd " | 3 | Church " | 2 | Folsom " | 12 |
| Perry " | 9 | Spring " | 9 | Pratt " | 6 |
| Sycamore " | 15 | Fourth " | 7 | Court " | 7 |
| Georgia " | 2 | Mechanic " | 2 | Lock " | 14 |
| Carroll " | 15 | Seventh " | 2 | Carolina " | 2 |
| Genesee " | 40 | Bennett " | 4 | Fifth " | 15 |
| Morgan " | 2 | Cherry " | 16 | Elm " | 37 |
| Sixth " | 3 | Moore " | 1 | Goodell " | 10 |
| Pine " | 3 | Louisiana " | 1 | Walnut " | 20 |
| Terrace " | 10 | Cedar " | 4 | Baker " | 1 |
| Ellicott " | I1 | Spruce " | 2 | William " | 13 |
| Main " | 8 | Allen " | 3 | Rock " | 50 |
| Ohio " | 8 | Water " | 13 | Washington " | 13 |
| Gay " | 3 | Pearl " | 4 | Green " | 8 |
| Chippewa " | 3 | Fulton " | 2 | Huron " | 3 |
| Hickory " | 4 | Cottage " | 1 | Exchange " | 46 |
| Carey " | 1 | Indiana " | 8 | Illinois " | 15 |
| Erie " | 2 | Bristol " | 2 | Ash " | 4 |
| Jackson " | 1 | Franklin " | 5 | Norton " | 2 |
| Evans " | 2 | Union " | 4 | Koon's Alley | 4 |
| Maiden Lane | 1 | Lutheran Alley | 4 | West side of canal | 3 |
| French Block | 8 | Sandy Town | 7 | Hydraulics | 35 |

The number of streets in which deaths, in a greater or less degree, occurred is, thus, 87, the whole number of streets in the city amounting to about 184. The reader conversant with the topography will perceive, that, while the disease prevailed in some streets much more than in others, the number of streets in which deaths occurred, and their situation relatively to each other, show that no area of much extent was entirely exempt from the disease.

The greater number of those affected with the disease, as in other places, were of the laboring classes, and by far the greater number, foreigners. This will appear from the hospital statistics to be presently submitted. It was observed that many persons of notoriously intemperate habits, and impaired constitutions, and who were looked upon as peculiarly susceptible, escaped, and that the great majority of cases were among persons who, from ignorance, or recklessness, resorted to no precautions, and neglected the preliminary symptoms.

At the first appearance of the epidemic no cholera hospital, as such, had been established. The Directors of the Buffalo Hospital of the Sisters of Charity promptly tendered to the City Council the use of that Institution to meet the emergency. In a short time, however, a building was fitted as a cholera hospital, to which all patients becoming a county charge were removed. We are indebted to the politeness of Dr. John S. Trowbridge, Health Physician, for the following statistics of the cholera hospital from the 8th of June to Sept. 10th, the dates of its being opened and closed:— The whole number received during the period just mentioned, was 243; of these, 112 were females, and 131, males.

The nativity of the patients received is as follows: Americans, 46; Irish, 110; Scotch, 8; English, 10; French, 5; Germans, 46; Hollanders, 6; Canadians, 2; Swedes, 2; Africans, 4; Swiss, 1; Norwegian, 1; unknown, 2.

Dr. T. states that of the above, 125 were received in a condition nearly, or quite collapsed, and some laboring under the fever consecutive to the disease. Many of the females died after premature labor. Several of the patients were discharged recovered, and afterward returned, having relapsed from imprudences; others relapsed while at the hospital, from imprudences, having eluded the vigilance of the nurses. At the first opening of the hospital the over crowded state of the wards was a serious obstacle in the way of a favorable issue of the disease. Afterward, this difficulty was obviated by the addition of temporary constructions, by which the accommodations were increased. In some instances, death occurred in consequence of the disease being combined with chronic affections.

No deaths occurred among those connected with the hospital as nurses, attendants, &c. Two female nurses, and one male attendant, however, experienced an attack, but all recovered. With these exceptions, all persons employed at the hospital enjoyed as good health, as persons generally who were disconnected with the establishment.

The number of recoveries at the Cholera Hospital was 115; number of deaths, 128.

In the success of treatment at the Cholera Hospital, as denoted by the ratio of mortality, it compares favorably with other institutions. For example, at the Charity Hospital of New Orleans, out of 1400 cholera patients, the number of deaths was 964. (*N. O. Med. & Surg. Jour.*) A large proportion of the patients were brought to the hospital in a stage of the disease almost hopeless in itself, and, as a general remark, they were of a class offering an unfavorable prospect for the successful operation of therapeutic measures under any form of severe disease.

The method of treatment pursued at this institution was uniform, and consisted of calomel and opium, with such other adjuvants as the peculiarities of each case appeared to require. The usual proportions of calomel and opium were, grs. xx of the former, to grs. ii of the latter, the same dose of calomel being repeated hourly, with a less quantity of opium, until an appreciable effect was produced, as denoted by a change in the character of the evacuations, and a corresponding improvement in other symptoms.

At the Hospital of the Sisters of Charity, 134 cases were received. Of these we will speak more fully presently. The number of patients treated at both institutions thus amounts to 377, being about one-seventh of the whole number of cases reported to the Board of Health.

The disease, near the close of the epidemic, was almost entirely confined to a particular section of the city, viz: the German settlements in the fourth ward. It lingered here for some time after it had mostly disappeared from other parts of the city.

It was observed, also, that toward the close of the epidemic, cases were more frequent in which the disease proceeded rapidly, in spite of remedies, to a fatal termination, showing a greater virulence than had previously been exhibited. This was apparent in our own observations, and was a matter of common remark among the practitioners of the city.

Anterior to the appearance of the disease in this city, and during the whole period of its continuance, disorders of the digestive system were extremely rife. The proportion of the population that entirely escaped more or less of these disorders was small. The disorders consisted in diarrhœa or looseness of the bowels, preceded and accompanied by lassitude, nausea, griping pains, sense of distension, and especially by borborygmus. The latter was an uniform and striking symptom. Usually, these symptoms were speedily arrested by simple measures. Occasionally, they were rebellious to medical treatment. Oftener they persisted in returning, again and again, so soon as remedial or prudential measures were intermitted. It was universally remarked that an amount of abdominal disturbance, such as other times would have occasioned no anxiety, and scarcely have claimed any attention, was followed by marked debility, patients recovering their strength slowly even after a slight attack.

Aside from cholera and the disorders just mentioned, few diseases occurred during the prevalence of the epidemic. The latter, and its allied disturbances, appeared to absorb all other affections. Cases even of cholera infantum, so common during the summer season, seemed less in number than usual.

As the epidemic declined, dysenteric affections became frequent. In some cases the latter form of disease was mild, and yielded readily to remedial measures, but in other instances it was severe, and occasionally proved fatal. It differed materially from ordinary sporadic dysentery. Painful tenesmus was less prominent, and the general or constitutional symptoms were disproportionate to the local evidences of mucous inflammation. The pulse, in severe cases, became very frequent, and prostration considerable; in some cases typho-mania was developed; in some, copious hemorrhage occurred from the bowels, and some patients fell into a state analogous to the collapsed stage of cholera. The two diseases, viz, cholera and dysentery, seemed, in some instances, to be commingled. We are unable to state what anatomical characters distinguished these cases, as no opportunities have occurred for autopsical examination. The dysenteric form of disease continued to prevail after cholera had ceased to be prevalent, but at this date, (Oct. 2d) it appears to have nearly disappeared.

As respects the meteorology of the period during which the Epidemic continued, we have nothing to offer beyond general impressions, no scientific observations on this point having been made within our knowledge. The season did not present any striking peculiarities in temperature, humidity, etc. Our impression is, that less rain fell than usual, and that greater extremes of heat and cold were experienced. So soon as the Epidemic ceased, it seemed to the sensations of persons directing their attention to this point, that some appreciable atmospheric change had occurred, more conducive to buoyancy of feeling. We were conscious, with others, of a decided alteration in the latter particular. It is, however, altogether propable that this is to be accounted for by the moral influence of the fact that so terrible a scourge had ceased its ravages.

With this brief historical sketch, we proceed to offer a few remarks, and present some farther facts, under the heads of the *Ætiology* and *Treatment*; afterward we will devote some space to the cases observed and treated by us at the Hospital of the Sisters of Charity, and in private practice.

The situation and climate of Buffalo are eminently salubrious. The greater portion of the city lies on a considerable elevation above the level of the lake. The soil generally is aluminous. A portion of the city, however, designated the "*flats*", on the borders of Buffalo creek, is low and marshy, and liable to inundations from the action of westerly winds.

In the latter locality miasmatic emanations are not entirely extinct. The air is pure, and, notwithstanding the proximity to the lake, by no means humid. Fogs are very infrequent. The extremes of heat and cold are not great, although the alternations of temperature are frequently sudden and considerable. During the last fourteen years (the period of our residence) no Epidemic disease has prevailed with much severity. During the prevalence of Epidemic erysipelas and puerperal fever in numerous sections of the country, this place did not entirely escape, but it suffered far less than other places even in the immediate vicinity. Scarlatina, during the period mentioned, has never prevailed extensively, or with malignancy. Typhus or typhoid fevers have never been rife. The ratio of ordinary diseases to the population, in so far as we may judge without any precise data for comparison, has been less than in eastern towns, for example, Boston, Mass. During the former visitation of Epidemic cholera, however, it suffered severely for two seasons, viz. 1832 and 1834. In the recent Epidemic it may be said to have suffered severely. The disease did not prevail to the same extent, or with as much fatality, as in some other western towns, viz., Cincinnati, St. Louis and Sandusky, but Buffalo comes next in order to these places, a greater number of cases in proportion to its size having occurred than in New York, Boston, and the intermediate towns. Now the question arises, what bearing have these facts on the Ætiology of the disease? We take it for granted that the existence of some Epidemic agency in the production of cholera is sufficiently established; in other words, that a special cause, irrespective of endemic causes, is involved in its development. The latter, however, may play an important part in originating and diffusing the disease; but their operation is auxiliary, and secondary to the essential cause, i. e.. the cause from which the disease receives its essential character. The important agency of endemic causes is shown by the fact that the disease is so generally confined to a small area, prevailing in cities especially, and frequently in limited sections of a city. It is not to be supposed that the special epidemic influence elects these situations to the exclusion of the vicinity, but it is there that auxiliary causes exist adequate, by cooperation with the special cause, to give to the latter requisite efficiency for the production of the disease.

In view of the salubrity of the climate and situation of Buffalo, we must look for some peculiar local circumstances operating in conjunction with the epidemic cause, in order to account for the prevalence of the disease. These circumstances probably relate to the character of a portion of the population of the city. The position of Buffalo is such, that it contains a large floating population, consisting of immigrants from all countries, in transition to places farther westward, and new comers of all kinds, seeking for occupation and new homes, in addition to the usual population of the laboring class, the destitute, and the devotees of vice. The class of laborers were greater than usual the present year, in consequence of the public works occupying a large body of men, mostly Irish and Germans. The various kinds of population just designated are peculiarly exposed to causes of disease. They are crowded together; many have recently endured the confinement, and privations, and sickness of a voyage across the Atlantic; not a few are impoverished, and the majority are desirous of consulting the strictest economy in their mode of living; they are strangers without homes, and often depressed by disappointments and anxieties incident to their position. Adding to these circumstances ignorance, recklessness, intemperance, and other vices, which will apply to a considerable number of the persons referred to, and we have sufficient reasons for regarding them as favorable subjects for any Epidemic disease like that under consideration.

The disease prevailed chiefly among the floating population of the city. We presume that we do not exaggerate when we say, that nine tenths of the cases were among the classes just enumerated. Nevertheless, a small proportion of the cases were among a different order of our population, to whom none of the foregoing considerations are applicable. How are these cases to be accounted for, especially when it is considered that in the neighboring towns no cases of the disease occurred (within our knowledge), except, in a few instances, persons were attacked who were lately from the city? We can only say, in answer to this question, that residence in a city, even under the most favorable circumstances, involves collateral causes favorable to the efficiency of the special cause of cholera, such as do not exist, ordinarily, to the same extent, in the country. The Epidemic influence was perceptible in the vicinity of Buffalo. Practitioners in the surrounding villages have informed us that intestinal disorder, or diarrhœa, was much more rife than usual, more intractable to remedies, and more likely to return. Why it should rarely, if ever, eventuate in cholera in the country, but occasionally so terminate in the city, the special influence being supposed to be alike in both instances, is, probably, susceptible of explanation by reference to the various circumstances to which all persons living in a city are exposed, which tend to impair the ability of the organism to resist an Epidemic disease. These circumstances we will not now stop to consider.

The question of contagion, which is still mooted, we will not discuss, but only direct the attention of the reader to the history of the Epidemic in Buffalo, as bearing on this point. It is obvious, from the relative situation of the first cases which successively occurred, and their relation in the order of time, that the disease could not have been transmitted from one person to another. The number of streets in which cases occurred, and that, too, simultaneously, and in rapid succession, is opposed to the idea of its having extended itself by means of a contagious principle. The fact that while more or less intercourse with the villages in the vicinity of Buffalo was kept up, and some cases occurring in persons coming from the city, no individual living in the country was known to have had the disease, affords negative proof against the doctrine of contagion. It will be conceded that the strongest evidence of the contagious character of an Epidemic disease, is derived from observations showing that a person coming from a section in which the Epidemic prevails, into a region where it is unknown, and there becoming affected with the disease, a greater or less number of those coming into contact with him become affected with the same disease, and it thus spreads over the neighborhood. This species of evidence of the contagious character of typhus has frequently been offered. The history of Epidemic cholera in this neighborhood, furnishes no such proof of its propagation by personal communicability.

Another method of proving the existence of a contagious principle, in any disease, is to determine whether the proportion of those brought into contact with persons laboring under the disease, and who become affected with it, exceeds, greatly, the proportion having the disease of those who are in no wise exposed. We have no data for exactness of computation, and comparison, on this point, as respects the Epidemic under consideration. We are sure that in a considerable number of the cases coming under our own observation, the disease could not be accounted for in that way, and, on the other hand, of those constantly in contact with cholera patients, so far as our knowledge extends, a very small proportion only experienced the disease. This fact, however, was noticed among the patients coming under observation, as will appear in the tabular abstracts of recorded cases accompanying this article, viz., several members of the same family were frequently attacked, either simultaneously, or successively. Of the cases occurring in our private practice, (ten in number), five occurred in two families, three in one, and two in the other; and in each family the several cases occurred at the same time, or with only two or three hours' interval. In such instances, of course, the idea that the disease may have been communicated from one to another cannot be entertained; but when cases succeed each other, or follow after a longer interval, it may seem to favor the supposition of a contagious principle. It is to be recollected, however, that different members of the same family are generally exposed, not only, in the same degree, to the epidemic cause, but also, in a like manner, to the various exciting, or collateral causes upon which the development of an attack depends. The latter consideration, it is believed, furnishes an explanation not less rational than the doctrine of contagion.

At the cholera hospital, as already stated, three cases occurred among the nurses and attendants, but it is to be borne in mind that the situation of a nurse, or attendant, in the crowded wards of a cholera hospital, involves a combination of auxiliary causes, which, acting in conjunction with the special cause, would be rationally expected to induce the disease in a certain proportion of instances. It would, assuredly, not be necessary to revert to the doctrine of contagion to explain the fact just referred to; still, if it were constantly observed that a certain proportion of those in close and continued proximity to the sick became affected, it would afford, it must be confessed, grounds for suspecting the presence of a contagious principle. At the Hospital of the Sisters of Charity, not one of the nurses, or attendants, experienced the disease. Owing to the limited number of wards it was not possible always to separate cholera from other cases. This was done in so far as it was practicable, in order to avoid unfavorable moral influences; but it was not always practicable. In no instance was the disease developed among the patients thus exposed. Three cases of cholera, however, occurred among the patients in the hospital. In two of these there had been no exposure. In the third case the person came with other members of the family laboring under the disease, and was attacked after her entrance.

Of the Physicians of the city, three suffered an attack, all of whom recovered. If we consider the labor performed by Practitioners at such a time, and the degree of anxiety incident to their duties, it certainly is surprising that so large a proportion escaped, shutting entirely out of view the question of contagion. One of the members of the Board of Health, (as already stated), Dr. Haddock, an educated physician, but not a practitioner, fell a victim to the disease. This event occurred early in the history of the Epidemic, and tended, in some degree, to foster popular apprehension lest the disease might be contagious. The circumstances connected with the event, however, were amply sufficient to account for it, without any reference to the doctrine of communicability. Dr. Haddock had been zealously and actively employed in the performance of his official duties, which were superadded to his ordinary business. He possessed an ardent and excitable temperament, and had become intensely engaged in the subject, and, moreover, persisted in his active, and exciting labors, after distinct premonitory symptoms had appeared, neglecting, also, timely resort to remedies. He died a martyr to his enthusiasm, philanthropy and public spirit.

As we have said, we do not intend to discuss the subject of contagion in this connection. We, therefore, make no reference to those considerations relating to the general history of the Epidemic, which, in our opinion, disprove the existence of a contagious principle as satisfactorily as any point is capable of being established by other than demonstrative evidence. We may, perhaps, at another time treat of this subject separately.

We shall offer no remarks under the head of the *Pathology* of the disease. We cannot profess to have attained any farther insight into the essential nature of cholera, by means of the ample opportunities for observation that have been afforded by the recent Epidemic, and our views, or rather prepossessions, on this point, have been already briefly explained. [See Vol. IV., page 504, No. for January, 1849.]

We pass, then, to the subject of *therapeutics*. Under this head we include, and will first offer some remarks on, the *prophylaxis*. Here, as in all other places in which this Epidemic has prevailed, the majority of the population experienced (as already stated) more or less disorder of the digestive system, giving rise to what are ordinarily called the *premonitory symptoms* of the disease. That they sustain toward the disease the relation of prodromes, is shown by the fact that in nearly all cases of the disease, they have preceded its development for a greater or less period. Now, with reference to these premonitions, the following important facts have been impressed by the experience of the late Epidemic in this city:— If they lead the patient to resort to proper prudential measures, and simple medication, cholera very rarely becomes developed. During the period that the Epidemic prevailed, we, as well as our brethren generally, were prescribing daily for great numbers of individuals affected with *premonitory symptoms*, yet we were so fortunate as to have but ten cases of cholera in private practice. Up to August 12th, we had had but three cases, in all of which recovery took place. On the 12th day of August, we had five cases, the attack in each occurring on that day, four of which ended fatally. To complete the statistics of our experience in private practice, of the remaining two cases, one recovered, and the other proved fatal. That the disease was averted in many of the hundreds of cases in which the premonitory symptoms were presented in varied shades of severity, we cannot doubt, although it is, of course, a matter not susceptible of positive proof. In but one of the cases occurring in our private practice, did the patient apply for the treatment of premonitory symptoms, and in the single excepted case, the only premonition was a slight nausea, without diarrhœa, or any intestinal disturbance. The premonitions in all but two, were slight, and of short duration. In so far as we are acquainted with the observations of our professional brethren, they corresponded with ours in this respect—*that scarcely in any instance in which premonitory symptoms received due attention, was cholera developed.* In the vast majority of the cases coming under our observation at the hospital, the premonitory symptoms had received no attention, either in the way of medical treatment, or ordinary prudence. This we know to be the fact, although we omitted to collect accurate statistics on this point.

These truths, in a practical point of view, are immensely important. It follows that the disease may, with great certainty, be averted in the vast majority of cases, if premonitory symptoms precede its development; and we know the latter to be the general law of the disease, although not without exceptions. Hence, a person, resolved not to neglect the earliest prodromic symptoms, but to resort to appropriate measures of prevention, need not entertain much apprehension of an attack of Epidemic cholera. These views are by no means novel. We offer the facts as observed in this city, only as tending to confirm their correctness and value.

It is worthy of remark, that very simple remedial measures, conjoined with due attention to hygienic regulations, will suffice for prophylaxis, when premonitory symptoms are present. This fact was exemplified at the U. S. recruiting Rendezvous of this city, during the period of the Epidemic. The recruiting party averaged from fifteen to twenty persons, consisting of a few stationary soldiers, the remainder being newly enlisted recruits. Recruits are often induced to enlist from destitution of other means of support, and they are apt to be given to dissipation. Many enlist in the sober period following excessive indulgence. From these circumstances, it would be supposed they would be good subjects for an epidemic disease, when it is also considered that, after their enlistment, before being sent to the recruiting depot, they have no duties to perform, and are exposed to all the temptations which a city like this affords. Moreover, the Recruiting Rendezvous is situated directly upon the bank of the canal, and in the immediate neighborhood several cases of cholera occurred. Not a single case, however, was developed among the recruits, or recruiting party, although almost daily some were affected with the premonitory symptoms. It seems fair to attribute their exemption to the means adopted for that object. These means were first, keeping the quarters cleanly, and directing any affected by premonitions to report to the sergeant, who was ordered by the commanding officer (Maj. Backus) to see that they directly took to the bed, and observed proper care in diet; second, a remedy was constantly at hand, consisting of equal parts of camphorated tincture of opium, and tincture of Rhatany, of which two drachms were given so soon as any symptoms appeared, and repeated at short intervals if required. An advantage of this combination, important to be considered in such cases, is, it is not unpleasant, either to the taste, or in its medicinal effects, and, hence, the class of persons for whom it was prescribed, would not neglect to apply for it from a repugnance to its administration. By pursuing this course, not only were no cases of cholera developed, but not in a single instance did diarrhœa, etc., require any other remedy. The simple combination employed, was dispensed, to some extent, among those residing in the neighborhood, and acquired considerable celebrity, within a small circuit, as an unfailing cholera preventive!

The ease and precision with which the premonitory symptoms were arrested by simple remedies, was a matter of common remark among the practitioners of the city. The difference in practice among different practitioners, consisted, chiefly, in the use of calomel in such cases. Some resorted to calomel in pretty large doses before any unequivocal choleraic symptoms were presented; others employed it under similar circumstances, but more sparingly; and others deferred its administration until cholera was actually present, or imminently threatened. Our own practice, in this respect, was to employ, usually, opium, stimulants, and astringents, alone, finding them sufficient. We were accustomed to prescribe opium in pretty full doses, say from 40 to 60 drops of the tincture, repeated at short intervals, until the diarrhœa, pain, borborygmus, etc., were arrested adding about the same quantity of tincture of camphor, and a little brand these measures, of course, being conjoined with rest in the recumbent posture, and abstinence for a short time. Other combinations, however, we frequently prescribed, such as the tincture of opium and camphor, and of Rhatany; chalk mixture combined with tincture of Rhatany, and camph tincture of opium; etc. The risk of needlessly producing salivation, is a sufficient objection, in our opinion, to the free use of mercurials in treating the premonitory symptoms, except unusually impending danger of cholera be present, inasmuch as experience shows other measures to be fully adequate to the end in view.

Great unanimity of opinion, and practice, among the Physicians of Buffalo, existed, as regards the general principles of treatment, when choleraic symptoms were present. Much reliance was placed upon calomel by all with whose views, or mode of prescribing, we became acquainted. Some diversity, however, existed, in the freedom with which this remedy was employed. By most practitioners it was given in pretty large doses, and repeated at short intervals—from twenty to forty grains, given hourly, until some change in the dejections, or other symptoms, was produced. The whole quantity thus administered, would average from one to three drachms. It was usually combined with opium, or morphia, and camphor, or capsicum, or both. As respects the proportions of the latter, different practitioners differed, some giving opiates and stimulants in larger doses than others. Diffusible stimulants were generally employed, in a greater or less degree, and here some difference in practice, as respects quantity, existed. Enemas of laudanum, or a solution of morphia, were generally employed, and frequently repeated, Some were accustomed to use, in conjunction, Tannin, and the acetate of lead, The acetate of lead, also, was frequently given in conjunction with opiates, etc., by the mouth. Suppositories of opium were sometimes employed. We cannot presume to give a more minute account of the practice pursued by our brethren here, based, as our knowledge is, upon information gained by casual intercourse. Our columns, of course, are open to any of them who will take the trouble to communicate a particular account of their methods of treatment, and the fruits of their observation and experience. We have no authority, nor disposition, to assume the position of reporter in their behalf. We will briefly state the course pursued in our own practice. Regarding the evidence in favor of calomel as too conclusive to be disregarded, we did not feel justified, generally, in withholding from those whose lives depended on our judgment as a practitioner, the supposed advantages of this remedy. In the quantity given, and the manner of giving it, etc., our course varied, and in a few cases, it was not administered at all. We have the same remark to make concerning the use of opium, or morphia. We seldom withheld thme entirely, but were guided by circumstances in the amount given. We were led by some of our early observations to think that the evils of giving opiates too freely, in some instances, are not inconsiderable. We think we have witnessed the occurrence of coma, and apnœa, when they were in part, at least, due to narcotism. So of diffusible stimulants,—

although we generally prescribed brandy in greater or less quantity, we have seen, as we believe, serious results follow excessive stimulation. We seldom, if ever, omitted, to prescribe camphor, and capsicum, regarding them as important elements of the treatment. External stimulating applications were, in all cases, resorted to, consisting of sinapisms, fomentations, or frictions with hot spirits of turpentine, and, in some instances, the hot mustard bath. The latter appeared sometimes to have a striking effect in producing reaction, but the difficulty of moving patients to, and from the bathing tub, is an objection which led us, after a time, to discontinue this measure. Enemas of Laudanum, or morphia, were seldom omitted, and we have thought that the addition of from ten to twenty grains of Tannin to each injection, sometimes rendered it much more effective. We employed suppositories of opium, in a few cases, apparently with good effect. To allay vomiting, the Creosote mixture was resorted to with success in many cases. This symptom frequently opposes a serious obstacle in the way of successful treatment, remedies being constantly rejected; hence, it is important, if practicable, to relieve it. Rigid restriction of drink seemed to us important to this end, in many instances. Ice, taken in small pieces, appeared sometimes highly useful, and, at the same time, afforded transient relief of the urgent thirst which usually attends the disease.

When the state of collapse was complete, we found pieces of unslacked lime, wrapped in moistened cloths, and placed under the bed clothes, to be the most efficient means of applying external heat. It is simple, readily available, and develops around the patient a heated atmosphere, the temperature of which may be elevated in any desirable degree. It fulfils all the objects to be attained by the heated vapor bath, without the inconvenience and delay of a more cumbersome apparatus. So soon as the condition of the stomach would permit, concentrated nutriment, conjoined with diffusible stimulants in moderate quantities, was administered, such as essence of beef, chicken broth, and milk porridge, with a view to renew, as speedily as practicable, the elements of nutrition lost by the serous discharges. Fluids were allowed freely when not contra-indicated by vomiting. The latter were regarded as important objects remaining after the first grand objects had been accomplished, to wit, to arrest the serous discharges, and obviate the immediate tendency to death. Other residuary objects of treatment appeared to be as follows:—1st, to relieve persistent symptoms, such as vomiting and diarrhœa. The former symptom was most apt to continue, the matters rejected generally being of a green color, as if tinctured with sulphate of copper. 2d, to obviate cerebral oppression, denoted by somnolency, gradually eventuating in coma, in which state several died, after the primary, urgent symptoms of the disease were relieved. The measures found most efficient for this object were, vesication of the nucha, and cold applications to the head. 3d, to restore the urinary secretion. The spiritus ether. nitrosus was uniformly prescribed for this end. 4th, to contribute to the restoration of strength by tonic remedies; and, with this view, Quinia was given, as early as practicable, in doses of two grains, every four or six hours. This was frequently combined with morphia, to relieve the irritability of the intestinal canal, which, in most cases, continued more or less, after the choleraic discharges have ceased. The latter often required, in addition, the continued use of anodyne enemas. In but a small proportion of cases did we think it necessary to employ cathartic, or laxative remedies, in any stage of the disease. Occasionally, however, it was followed by a degree of constipation which required attention, and the article prescribed, in such instances, was rheubarb, sometimes conjoined with simple enemas.

The foregoing is a cursory view of the principles of treatment followed in the cases coming under our charge. We have already presented the statistics of our cases in private practice. It remains to speak of the cases received at the Hospital of the Sisters of Charity, treated by us, as the attending Physician to that Institution.

The whole number of cases received at this Hospital, up to Sept. 1st, is 134. Of this number 52 were fatal. This makes a ratio of one death to a little over two and a half patients received. The mortality, as the reader will perceive, exceeds slightly that of the whole number of cases reported to the Board of Health, in which the ratio of deaths was as one to a little less than three. A small number of the cases at the Hospital were treated by other Physicians, being private patients; and a few were sent to the Institution by the Board of Health, and attended by the Health Physician. About one hundred and twenty-five were treated by the attending Physician of the Hospital.

In registering patients as cases of Epidemic cholera, the principles of diagnosis, as adopted by the Buffalo City Association, in a report published in the August number of the present volume of this Journal, were rigidly followed. No case was registered as cholera, unless choleraic discharges were observed, or a combination of symptoms presented, which rendered the diagnosis positive. A large number of cases were received as cases of cholera, which were not so registered, because adequate evidence of the disease was not determined. These cases were registered as cases of diarrhœa, many of which were severe, as the fact of their coming to the Hospital suffices to show. Thirty-nine cases of Diarrhœa appear on the Hospital register during the half year which includes the period of the Epidemic up to Sept. 1st. The greater proportion of these were cases of cholerine, or of the premonitory symptoms of cholera unusually severe or persistent.

Of the unequivocal cases of cholera received, a large number were already in the stage of collapse. Many died very soon after admission, and several never came under the observation of the attending Physician, the patients entering, and dying, between the hours of his attendance.

Considering the character of Hospital cases, the results of the Charity Hospital, as declared by the rate of mortality, certainly affords grounds for much satisfaction. We do not hesitate to use this language, because we assume no special merit on the score of the medical treatment, which, so far as we know, was essentially similar to that pursued by many, if not by most of our professional brethren. Stating this, in all frankness and sincerity, we trust we shall not expose ourselves to any imputations of indelicacy, from our personal connection with the Institution. We are free to say, that whatever credit is due to the Institution for the large proportion of recoveries, belongs to those under whose immediate charge the Institution is placed. Much advantage, also, accrued from the fact that, at no time, were there collected together a very large number of cholera patients. If many cases of this disease are simultaneously received at a public Institution, such is the urgency of the symptoms, and so great the necessity of promptness in the administration of remedies and other measures, that, unless the number of attendants is very large, and their fidelity and intelligence to be relied upon, some of the patients will receive insufficient attention, and injustice will not fail to be done to the resources of medical art.

Each patient admitted at the Hospital, was, at once, placed under the charge of one of the Sisters, and received her unceasing and assiduous care so long as it was requisite. Scrupulous exactness in the execution of all medical directions, and fidelity in the administration of remedies, could be confidently depended upon, together with all other attentions, and appliances, which the circumstances of the case might suggest. The degree of patience and endurance exhibited by the Sisters of Charity in their unwearied labors of mercy, during the period of the Epidemic, was a matter of astonishment not less than admiration. Night after night, as well as on successive days, they were at their post, never manifesting weariness, or diminished zeal; and during the whole period, not one was debarred, by illness, from the exercise of her voluntarily assumed duties. We would not be thought to utter a common-place sentiment, and we hope it will not be deemed irrelevant, in a paper of this description, when we express the belief, that a signal instance was here afforded of strength vouchsafed beyond the unaided resources of the physical and moral constitution.

Of eighty of the cases of Epidemic cholera coming under our professional charge, in Hospital and private practice, we have preserved notes; but owing to the extraordinary claims upon our time and strength, in several instances they were very imperfectly made, and, in general, they are far lesss complete than we could desire. We were not careful, always, to record all the phenomena present, and, also, to note what phenomena were absent; hence, much to our regret, we have not data sufficient for an accurate enumeration and comparison of the events which belong to the descriptive history of the disease. No leisure was afforded for autopsical researches; but this is less to be regretted, inasmuch as the annals of medical observations are not deficient in facts pertaining to the appearances found after death.

In order to make our records in some slight degree available, or, at all events, for our own satisfaction, we have analyzed each case with reference to the facts noted, and arranged the symptoms under separate anatomical divisions. The results of this analysis are submitted in the tabular abstracts accompanying this article. Out of the eighty cases, thirteen were found too defective to possess any value. The remaining sixty seven cases are distributed into two classes, from the result being in death or recovery, and a tabular abstract is devoted to each class.

To avoid misapprehension, we would again state, that the histories of the cases analyzed, are not sufficiently minute and comprehensive. Pains were not taken to note the presence, or absence, in many cases, of symptoms which were almost invariably present, i. e., the suppression of urine, restlessness, thirst, etc. Still the tables exhibit a rough sketch of the events which constitute the historical features of the disease, and will enable the reader to form some idea of their mutual relations, and the relative frequency of their occurrence. He will also be able to institute some comparison of the two classes of cases as respects the symptoms and treatment.

We had designed to have given, by way of conclusion of this report, a few selected cases in full. The article, however, is extended already so much beyond the limits contemplated when it was commenced, that we must reserve the execution of this purpose for another number, or postpone it indefinitely.

BUFFALO, Oct. 8th, 1849.

